# Feedback gap and strategies for handling criticism in early surgical career

**DOI:** 10.1016/j.sopen.2025.11.005

**Published:** 2025-11-21

**Authors:** Hanne Pedersen, Alexander Tejera, Christopher Mathieu, Britt-Marie Johansson, Magnus Anderberg, Kristine Hagelsteen

**Affiliations:** a Lund University, Department of Clinical Sciences, Pediatrics, Lund, Sweden; b Practicum Clinical Skills Centres, Skåne University Hospital, Lund, Sweden; c Lund University, Birgit Rausing Centre for Medical Humanities, Lund, Sweden; d Lund University, Faculty of Social Sciences, Department of Sociology, Lund, Sweden

**Keywords:** Feedback, Feedback culture, Learning environment, Resident, Surgery

## Abstract

**Objective:**

The aim of this study was to explore experiences and challenges in handling feedback and criticism among early career surgeons.

**Design:**

This study is part of a prospective, exploratory, longitudinal study evaluating surgical residents throughout residency. Semi-structured interviews were conducted with medical doctors applying to a locum or residency position in a surgical specialty. Analysis was performed using a cross-sectional thematic analysis.

**Setting:**

Departments in general surgery, urology, and pediatric surgery at seven hospitals in Sweden.

**Participants:**

Contact information to applicants interviewed for a locum or resident position at the included departments were forwarded to the research group. The research group contacted applicants for inclusion and 50 were included.

**Results:**

Four themes were constructed in relation to the participants' management strategies and experiences: 1) reflection and processing of criticism, 2) emotional response to criticism, 3) cautious feedback culture, and 4) navigating criticism in a hierarchical system.

**Conclusion:**

This study revealed barriers to an effective feedback culture in a pool of applicants for a residency or locum position in surgical disciplines. A culture of reluctance to give feedback was a strong and common denominator. A process of filtering feedback could possibly be a method of survival and thriving in the contemporary workplace. Suggestions and initiatives to change the feedback culture are proposed.

## Highlights

• Applicants for a surgical residency position described a deficient feedback culture.
• A reluctancy to give feedback was a strong and common denominator.
• Unfair critique is a part of the learning environment.
• To filter and analyze feedback could possibly be a method to survive and thrive.

## Graphical abstract

**Figure:**
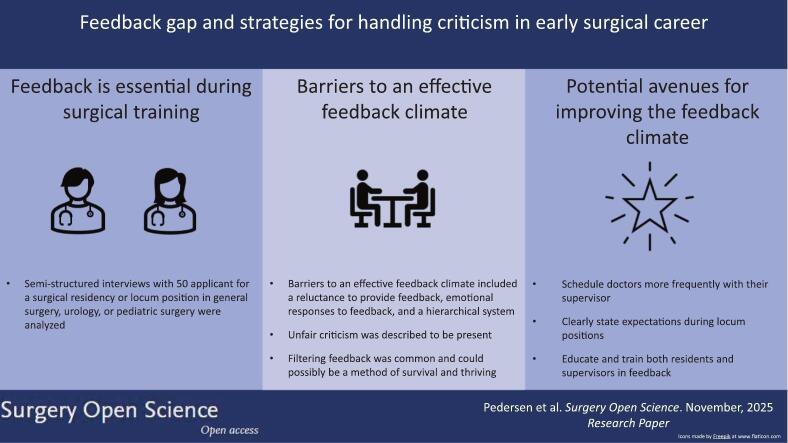

## Introduction

During surgical training, residents need to develop a wide range of both technical and non-technical skills to become competent surgeons [[1], [2], [3], [4], [5], [6], [7], [8]]. With the transition towards competency-based medical education, focus has shifted from less well-defined outcome measures in a time-based training model to evaluation of acquired competencies and a more flexible timeframe [9,10]. Modern residency training and curriculum design are based on multiple colleagues giving residents formative feedback on all aspects of the professional role to develop into competent specialists in the preferred field. The feedback should be conducted and delivered in a structured fashion and based on observations. In Sweden, residency training is based on guidelines from the National Board of Health and Welfare, and from these guidelines the trade union (the Swedish Medical Association) have developed more detailed recommendations for structured evaluations [11]. These guidelines include both suggested frequency and methods for evaluations during surgical training. Continuous assessments of both technical and non-technical skills with validated instruments are recommended, using for example Case Based Discussion or Mini Clinical Evaluation Exercises [12].

Feedback implies a low-stakes situation and differs from assessment by being formative instead of summative [13]. Formative feedback is essential for learning in the competency-based model [10] and both the quality of feedback given to the residents and the learning environment play a crucial role [14,15]. The workplace's learning environment is important for resident development [16], and a lack of feedback impedes progress and development. A lack of structured, timely, specific, and regular feedback has been previously described in several countries among residents, including Sweden [[17], [18], [19]]. According to Hattie et al., both positive and negative feedback can be effective in stimulating learning [20]. However, in a study on medical residents positive feedback led to positive feelings among most participants, while negative feedback was commonly associated with feelings of embarrassment, stress, or humiliation. Additionally, one third further described the fear of receiving negative feedback as a barrier to feedback-seeking [18].

In medical education research, the focus is often on specific types of or different models of communicating feedback and how to make it effective. Many clinicians are not familiar with these theoretical models or frameworks, and a considerable terminological ambiguity and lack of stringency in language can surround the use of the words “feedback” and “criticism”. McCarthy et al. state that this terminological confusion also exists for the terms “feedback” and “assessment” [21] which are frequently used interchangeably in competency based medical education.

In Sweden, early evaluation of young medical doctors is of additional interest since the first 6–12 months of residency often consist of a locum position. The recruitment for the locum and resident positions is by tradition decentralized and based on a department's local needs. It is not structured to a particular time of year, there are no specified guidelines, and the current practise is an *ad hoc* interview and some reference taking before gaining employment. The idea behind this system is that both the surgically interested individual and the employer can explore the individual's aptness for a surgical career under a specified timeframe [22]. The time as a locum is therefore dependent on continuous evaluations from employer and colleagues to assess future potential, however, there are no clear guidelines for these assessments. The employers trust their colleagues to observe and report about the newly hired individual. Further, there is an ongoing reformation of the educational system with the addition of an extra semester to undergraduate medical training. When implemented, medical students will become fully licensed physicians immediately upon graduation, while the previous system included an internship of clinical rotation work, including surgery, for 18–24 months before becoming licensed. This new system implies shorter clinical experience when applying for a residency position and highlights the need for extensive feedback early in the career [23,24].

Overall, feedback is an essential part of residency training, and previous research has described deficiencies in, for example, frequency of feedback. To further investigate potential barriers, this study focuses on the experience of feedback of applicants for a residency or locum position in three surgical disciplines. The aim of this study was to explore experiences and challenges in handling of feedback and criticism in early career surgeons.

## Method

### Participants

This study is part of an ongoing longitudinal prospective study repeatedly assessing residents during surgical residency. In total 73 applicants to surgical residency positions in general surgery, urology, and pediatric surgery at seven hospitals in Sweden were invited and 50 managed to be scheduled. Forty-three participants started a position as a resident (*n* = 34) or locum (*n* = 9). See Table 1 for descriptive characteristics. The inclusion period spanned from 2017 to 2021, encompassing both regional and university hospitals. This cohort has previously been described [25,26]. The hiring departments provided the participants´ contact information to the research group for those called for a job interview to a locum or residency position. The research group invited all the applicants to participate in the study and scheduled those accepting participation for a full day of tests and interviews at the Skåne University Hospital's Clinical Skills Centre in proximity to the recruitment process. The interviews and tests for the study were always performed after the decision on employment to either a locum position or residency had been made by the hiring department, and in some cases participants were not offered employment. Participants were requested to submit a background form, which inquired about various demographic and professional characteristics, including age, sex, county of education, job experience, current position, and the department to which they sought employment. Background forms were collected and managed using REDCap electronic data capture hosted by Lund University, Sweden [27,28].

**Table 1:** Background variables for participants in the study.

Background variables		n
*Sex*	Woman	20
	Man	30
*Age, median and range (years)*	30 (27–41)	
*Country of medical education*	Sweden	31
	Other Nordic country	6
	Other European country	13
*Specialty*	General surgery	34
	Urology	15
	Pediatric surgery	1
*Employment*	Resident	34
	Locum	9
	No employment offered	7
*Previous specialist in another field*	Yes	1
	No	49

### Interviewing

Semi-structured interviews were held in person and performed at the Practicum Skills Centre in Lund during a test day. An interview guide developed during a research study focusing on potential “warning signs” among surgical residents was used. The interview guide has been previously published and comprises of 26 questions, employing neutral or positive phrasing (Appendix 1) [29]. The interviews were conducted by one or two experienced sociologists (BMJ, CM), most often with assistance from an experienced surgeon. All interviews were conducted in Swedish. Participants were deidentified using a study-ID and asked to describe situations and events without identifiable markers such as cities or names. The interviews were audio recorded with the consent of the participants and stored on password-secure external hard drives and at LUSEC, a platform hosted by Lund University that fulfills all demands of safe storage of research data. One audio-recording was incomplete due to technical malfunction, resulting in the loss of answers to the final seven questions. The study-ID was simplified to a number for use in this manuscript.

### Qualitative analysis

Verbatim transcriptions of the interviews were made by medical secretaries or students involved in the research group and imported into NVivo (Lumivero, Version 14). A cross-sectional, thematic analysis was performed. Two researchers (AT, HP) separately coded the transcripts focusing on the semantic meaning expressed by the participants. Coding was performed deductively where statements from the participants were assigned to previously identified “warning signs” [29]. Following the preliminary coding, a unified coding document was created, and the sorting of data extracts to warning signs were discussed between HP and AT until consensus was reached. To further enhance and develop the analysis, the unified coding was discussed with CM, BMJ and KH and interviews were revisited. From the sorted codes, HP developed themes focusing on participants' handling of feedback and criticism. These themes were constructed more inductively without regard to which warning sign the underlying data extracts had been originally sorted. For this paper, due to conceptual overlap, the terms feedback and criticism are used synonymously in the analysis of the interviews. Themes were repeatedly discussed within the research group, and the interview transcripts were revisited to ensure representativity until the manuscript was finalized.

### Reflexivity and ethics

HP and AT are medical doctors and PhD-candidates in medical education specifically studying aspects of surgical education. In their clinical work as residents (HP in internal medicine and AT in pediatrics) they have no further connection with the context studied. The senior author KH is a senior consultant in pediatric surgery practicing at Skåne University Hospital (Sus), one of the hospitals included in the study, and has previously been program director for the pediatric surgery residency program as well as director of postgraduate training at Sus. She thus has an inside perspective on surgical residency training in the context of the study. MA is the head of department in pediatric surgery at Sus and conducts recruitment to pediatric surgery residency. None of the participants included in this study were included from this department. CM and BMJ are both sociologists at Lund University with an extensive background (+20 years) in qualitative research studying the sociology of work. They bring an outside perspective of the workplace training in the healthcare system. Working together as an interdisciplinary team having continuous discussions, as previously described [30], helped us to bring our individual preconceptions to the spotlight, explore these from different perspectives and reach a deeper understanding of both the context and data of the study.

Ethical approval for the longitudinal research project, including the study of this article, was obtained from the Swedish Ethical Review Agency (approval number 2016/1050, with amendments 2022-04361-02, and 2023-04850-02). Informed written consent was obtained from all participants.

## Results

From the sorted codes, four themes related to feedback and criticism were constructed in relation to the participants' management strategies and experiences: 1) reflection and processing of criticism, 2) emotional response to criticism, 3) cautious feedback culture, and 4) navigating criticism in a hierarchical system. The themes are presented together with descriptions of perceptions and examples of lived experiences from the participants and covers management and challenges regarding work-related feedback. Table 2 illustrates examples of quotes for each theme. The examples brought up were not always taken from surgical departments and could refer to the participant's previous working experiences.

**Table 2:** The four themes related to feedback and criticism identified from the interviews with examples of quotes regarding each theme.

Theme	Examples of quotes
Reflection and processing of criticism	“I listen to it, to the whole situation, and then analyse my behaviour. If it is an experience that the person has been part of then that experience is true for that person and then I have to apologize for the misunderstanding. If it is something I have done wrong, I usually feel that it was my fault. But sometimes when criticised by patients I have thought it through and discussed it with a supervisor and felt like I should have done it in the same way next time. Yes, one part might have been my fault but not another one, and I have to listen and learn, to get criticised but not let it get to me that I have done something wrong if a colleague can support the medical decision. But it is much about communication, it can be that kind of flaws.” (IP 7)
	“I think you should accept all critique, but you don't have to implement it if you don't fell it is reasonable. The times I have been criticized I have listened to it but it is not always you find it fair, perhaps I was stressed or had other reasons for my actions. But the times you feel it is constructive you have to be open for it, we are not perfect.” (IP 11)
Emotional response to criticism	“Or colleagues, it can also be colleagues that yells at you for quite bizarre reasons, when you don't really understand. Then I can doubt what I am doing and doubt myself.” (IP 30)
	“Yes, it is hard to say, I take it with me. I might remember things a rather long time, my mom always nags on me for this. I am usually good at remembering the negative and bad at remembering the positive. It depends on what it is, of course it is never fun to receive negative criticism but at the same time you have to take it and make something good of it.” (IP 14)
Cautious feedback culture	“How do I do, good question. I think I in general, as everyone else, think it is hard to provide critique. But I usually say, in this situation I saw that you did this, and what you can think about in the future is to try this other way.” (IP 1)
	“I don't like to step on anyone's toes, but if I think something is wrong, I say it sometimes, but not always. I try to be rather diplomatic.” (IP 42*)*
Navigating criticism in a hierarchical system	“It depends on, unfortunately it is not easy, if it is a senior doctor higher in the hierarchy, to criticize them, at least not at our clinic. So it depends on how receptive that person is and how urgent it is. If it is something dangerous of course I will act, but if it has something to do with that there are different styles, styles of treatment, and we don't agree with each other then it is just an opinion, and it depends. But unfortunately, it is not that easy and even if it is constructive criticism to someone senior, not everyone is receptive, all though some are.” (IP 31)
	“But like I said before, because there are hierarchical structures in healthcare, it can be hard to discuss with a consultant. If it is pure discrimination and things that happen between colleagues then I try to bring it up with a superior or question in the ongoing situation. It depends on what has happened. I have for example when I have felt suppressed a consultant emailed him. And I told him how I felt and that I thought it is important with respect. Interviewer: Did you get a good response? IP: No, something like have a good life. But it still felt right, I think it is important to stand your ground.” (IP 13)

### Reflection and processing of criticism

In the interviews a process of reflection and analyzing criticism was commonly described. This analysis led to either accepting or rejecting the criticism, depending on if the participant agreed with the criticism and description of the situation, or found it unreasonable:

“*I usually don't just accept the feedback as it is, because even if people are usually right, but not always, you have to think a little bit to integrate into your own world.*”

(IP15)

Occasional descriptions from the participants were made of discussing the criticism later with a supervisor or a friend, suggesting an even longer period of reflection. A reason for the criticism to be perceived as unreasonable could be that it had been given around a situation impacted by other factors, such as stress, that the participant felt could explain or excuse the behavior. Other examples were when there was a mismatch in the belief of whether it regarded an actual problem, such as spending too much time on a patient, or preferences for different methods, or if the criticism was presented in a judgmental or angry manner which made it harder to accept and use for self-improvement.

Some participants did however state an openness towards criticism, and a willingness to improve, even though it was affected by the mode of delivery:

“*I usually always accept and like criticism if it is delivered in the right way because it is an excellent opportunity to learn and improve.*”

(IP 37)

### Emotional response to criticism

Some reported an overall sensitivity and difficulties handling critique, as well as a work environment where unfair criticism was present. This sensitivity was characterized by internalization and subsequent contemplation and occasionally leading to self-doubt. The participants described different responses and reflections to these feelings, from trying to improve to being more cautious around the person that provided the feedback or around similar emerging situations. Receiving criticism was perceived to be two-sided, as one participant described a feeling of embarrassment when being criticized, but when not having received any feedback at all instead was feeling insecure and contemplating whether the performance had been good enough:

“*I think I ponder before, which might not be optimal. But it can be liberating if someone says you should have done like this instead. So, you get feedback and do not walk around wondering if people think you are bad. I think I more often bring work home and think if I don't get any feedback than the other way around.*”

(IP 16)

While often contrasting with a usual openness for criticism, there were descriptions of receiving unfair criticism which revealed different approaches among the participants. Some got irritated, but descriptions ranged from being unaffected to serious self-doubt in the professional role.

“*If it is given in a very condescending way, very patronizing and mean, without any reason that could help me move forward, then I get irritated rather than accept it.*”

(IP 38)

Not only personal factors were brought up as problematic. The situation was sometimes added as having additional impact on what was perceived as an already unfair criticism. One participant described a situation of being pointed out and criticized in a meeting with several other doctors, despite calling one of them to ask for help a few minutes earlier and being rejected due to lack of time:

“*at that moment you feel really bad, like why did I become a doctor? (…) You might not always think of how you provide criticism, and sure, you can understand that perhaps you don't have the energy to sugarcoat it and instead it gets very straight. On a good day I can handle it, but on a bad day it isn't good when I get home.*”

(IP 4)

Several participants brought up aggravating difficulties in handling critique due to a work environment where unfair criticism was present. Consequently, their response to critique differed depending on context or mode of delivery. Some described examples of being yelled at for unfair reasons, such as being criticized for situations that had not happened or where a relative of a patient was angry. The experience of unfair criticism could even lead to change in career path, where one participant described having left a career in research:

“*I had the opportunity to visit another group, and I saw that they had this great equipment, and I hadn't had any results, every measurement was different. I told the research group leader, who scolded me for not finding my own solutions and being more creative, and for always relying on money. It was upset because I did not expect to be yelled at in front of the whole research group, (…) and then I quit after many years of research.*”

(IP 9)

### Cautious feedback culture

Several participants described a learning environment where cautiousness or unwillingness to provide criticism to colleagues was present. This regarded both feedback concerning themselves and the ones they supervised.

One participant who had not received a residency position stated that he/she had not received critique or constructive feedback during the locum time, and therefore not had been able to correct behavior. A few participants with prior experience from working abroad experienced the Swedish work culture as more cautious with criticism and feedback in relation to their previous experience from other European countries. Furthermore, these participants had noted a general absence of feedback in the Swedish work environment, and that the responsibility for obtaining feedback was the junior doctors' responsibility and nothing to take for granted or expected.

“*In my experience, you don't like to criticize others here, and you try not to. You might not like it, but you don't say anything. I would say it happens much more in [country] that you get feedback, often on things that were not good, and you have to deal with it because it is not always in a way that is easy to receive. But I think you get more feedback and a better understanding of what you did right and wrong, and here [in Sweden] you have to ask. Did I do a good job, or could you give me some feedback so I know? What can I do better?*”

(IP 17)

Several participants expressed difficulties providing feedback themselves, and some clearly stated a cultural influence for this behavior:

“*I am trained in the Swedish health care system, so I am not great at giving criticism*.”

(IP 40)

A difference was described in that positive feedback and suggestions for improvements were regarded easier to provide than negative feedback. Some participants expressed a general fear of being part of, or initiating, a conflict by expressing criticism, affecting the will to speak up or taking fights even though the fights were deemed to be adequate.

### Navigating criticism in a hierarchical system

The participants clearly perceived that they were working in a hierarchical system that exhibited challenges regarding criticism and education. There were several descriptions of differences in the participants' approach to giving feedback or critique depended on if it was directed at a supervisor or someone they tutored.

“*…if it is someone superior to me doing something wrong, I think I just ignore that person and do what I think is right and change without them noticing. But if it is someone lower in rank it is often due to inexperience and then I might be more verbal in telling them what needs to change*”

(IP 24)

Justifications for this kind of behavior included respect for hierarchal structures and authority as well as fear of retaliation. A few participants described that rather than discussing the matter with their superior, they corrected mistakes in secret. To avoid conflicts, some participants stated taking potential problems or witnessed mistakes to a higher level of management and engaging in discussion with the department head instead of facing their supervisor or colleague themselves. A few expressed a feeling of resignation to hierarchical structure and that ultimately the responsibility lies with the senior colleague even though a mistake was apparent. In other instances, both severity of the error and hierarchy were considered when reflecting about acting or not:

“*It is a conflict; on the one hand there is the element of fear, and on the other hand there is the seriousness of the mistake. If the mistake is serious, I have to overcome my fear. You don't criticize your boss for a minor event, but if it's someone you're teaching you can give them feedback.*”

(IP15)

A common solution to ambiguity in such situations was to rephrase potential critique as a question directed at a superior in the hierarchical structure. This approach involved either feigning a lack of knowledge or providing examples of how others have previously conducted the same procedure or handled an event. These methods were purportedly effective in empowering the role of the individual in an educational position in relation to the supervisor. Conversely, the provision of suggestions for improvements was advocated when the participant was in a supervisory position.

Some described greater difficulties to comment on behavior than on medical or technical mistakes:

“*(…) when I have heard colleagues talking to patients, which is the most challenging thing, it does not sound good. You can't say that. Unfortunately, I wasn't able to tell the colleague that you can't talk like that, because it was colleagues that I have felt I couldn't criticize without getting into trouble*.”

(IP 9)

or

“*Behavior is probably harder. It is much more sensitive. I don't know what I would do if an older colleague behaved really badly towards someone. That would be a really difficult situation.*”

(IP 40)

## Discussion

The participants described a clinical workplace environment characterized by difficulties in handling criticism and insufficient feedback. They attributed these challenges to a mixture of personal, structural, and hierarchical factors.

Some participants described a lack of feedback that does not meet the demands of a modern competency-based education model. The described absence of feedback impedes progress and development, and the lack of regular feedback is a known problem area even in other countries [[17], [18], [19]]. In this study, participants with experience from other countries described an even more cautious approach to feedback and criticism in Sweden, suggesting this might be an even larger problem here compared to other countries. Given the experience from one of the participants who did not receive a residency position, who stated to have received no feedback that things weren't progressing as expected, it might be that neither feedback, nor what the expectations are, is communicated clearly when being employed.

The experienced lack of feedback might in part be due to misaligned expectations between the newly employed and the senior colleagues. Other studies have shown a mismatch in the perception of quality and frequency in feedback between learners and educators [19,31,32], and this has been suggested in part to be due to the failure in recognizing feedback by the learner [13]. The contradictory expectations of feedback, enhancing mutual understanding of its conceptual underpinnings, and training of assessors, have been posited as avenues for potential educational improvements [19,32]. An additional factor that impedes the possibility for feedback and development among residents is a system where residents often are scheduled to work with many different colleagues over a short period of time. This scheduling leads to fragmented supervision and affect the teacher-learner relationship in a negative way [33]. It has also been described as a barrier to feedback by senior surgeons by Hagelsteen et al. [29] Further, in Sweden, residents are expected to take on large responsibilities in the emergency departments, often working with limited supervision and thus low potential for feedback from direct observation by colleagues. An even more aggravating finding is the fact that the participants in this study themselves demonstrated behaviors concordant with their supervisors, thereby sustaining the current culture stating difficulties in providing feedback to colleagues. On the positive side, some of the participants described a willingness to engage with constructive feedback for development in their supervisory role. This should be recognised and encouraged early to enable a sustainable cultural change.

Different approaches for the management of feedback and criticism were delineated in the data and revealed a common strategy to process, filter and analyze the feedback before eventually accepting it. Their strategies largely affected the participants, where one made decisions to a change career path after heavy critique, and some decided to ignore it. In the interviews there was an interchangeable use of “criticism” and “negative feedback” which contribute to a conceptual ambiguity. This could perhaps be due to unfamiliarity with the theoretical concepts, or a similarity between the two, with the fact that negative feedback could lead to feelings of embarrassment and stress [18]. It could be argued that working in an educational position under supervision and not accepting criticism is arrogant or a sign of hybris, according to Hagelsteen et al., a so-called warning sign or red flag [29]. However, with the consideration of descriptions of mean and unfair critique, it could instead be argued that filtering critique is a method of survival and thriving in the contemporary workplace. It might be that the adoption of this mindset could enhance an individual's resilience to manage errors and maintain motivation when facing personal setbacks. On the other end of the spectrum, some of the participants mentioned being sensitive to criticism and internalizing it. This sometimes affected them both concerning insecurity as well as physical and mental wellbeing. If developing self-doubt, the potential for improvement could speculatively decrease, as well as increase the fear of additional critique. The fear of criticism has been described a barrier to feedback-seeking [18], which in turn would impede development. Another potential outcome would be the need for even more feedback or support to progress as remediation efforts might be initiated [34]. Not solely personal factors were brought up as problematic in handling of criticism, but the context of a situation, such as being pointed out in front of others, also affected the response. The internalization on feedback might be indicative of adverse effects on their well-being. It is plausible that the sensitivity to critique is partly related to limited work experience, being low in rank in the hierarchy, and might diminish with time. Either way, it is important for supervisors and educators to be aware of this filtering process and the possible emotional responses to feedback. Explicitly discussing this process with learners can help improve the feedback culture and reduce barriers to effective feedback.

The underlying hierarchical system and described fear of retaliation may further impact the sensitivity to feedback or criticism. A fear of retaliation and involving in conflicts might be adequate, since 17 % of Swedish doctors in a survey stated to have been punished for criticizing their workplace [35]. The Swedish culture is in many ways considered an equal and non-hierarchical society, but the experiences from the early career surgeons in this study clearly witness that the hierarchy in surgical departments is still present.

A challenge with the current system is reflected by the participant stating that they only have received feedback in relation to applying for a permanent residency position, and not for improvement working as a locum. This is perhaps even more problematic since evaluations have obviously been conducted but the person affected has not been informed, or at least not to the point of understanding. Without feedback for professional improvements the value of the work trial period diminishes. Furthermore, the potential for development and accurate evaluation is reduced.

According to the findings in this study, suggestions that could address the issues raised concerning lack of systematic feedback provided by more experienced colleagues on an organizational level are proposed. A change in the current work climate to improve the feedback culture would need to focus on actions and mental models to mitigate how criticism and feedback are delivered. These actions cannot directly impact how feedback is perceived by the receiving locum or resident but hopefully contribute to a better learning environment.

First, avoiding fragmented supervision by scheduling new colleagues to work on a regular basis side by side with their supervisor could facilitate observations of situations suitable to give feedback on, and over time it could improve work culture. Working close together would allow colleagues to bond and hopefully create a safe work environment at a low cost.

Second, clear communication from decision-making stakeholders to locums and new residents concerning expected goals and competencies to reach during the locum period or first 6–12 months. These goals and competencies need to be assessed in a structured manner, with backup “action plans” if the locum or resident does not manage to “level up to expectations” [34]. With the implementation of the changes in medical school in Sweden leading to decreased clinical experience before starting residency, early feedback for development will likely be of even greater importance. This might seem easier than done, and in many parts of the Western world, competency-based medical education has been introduced in postgraduate training with the explicit focus on both assessment for learning and assessment of learning [36]. Compulsory assessments in line with a competency-based education could be one way forward to improving assessment of residents. However, this also comes with challenges, and a recent debate has highlighted assessment burden as a problem within competency-based postgraduate training [37,38]. Therefore, care needs to be taken so that assessments are not to be viewed as an administrative burden but instead support learning for residents. Though, at the moment, the current learning environment is far from being overloaded with assessments, like the descriptions mentioned above.

Third, education and training on all levels, *e.g.* the residents, supervisors and senior colleagues, in feedback and assessment to improve their knowledge in the field and make the scientific underpinning behind assessment and feedback as valuable to them as clinical guidelines are concerning diseases to treat. This might lead to alignment in views on feedback and assessment language and interpretations as well as delivery. Initiatives to support this kind of change have been started and needs to be disseminated in the medical society [39].

Fourth, constructing a system where “everyone” gives feedback to “everyone” in the hierarchy could reduce the tension of giving and receiving feedback, and reduce hierarchical power relations. Assessment instruments available are for instance “The Maastricht Clinical Teaching Questionnaire (MCTQ)” [40], which could be used by residents conducting “faculty meetings” with discussions concerning experience of supervision with structured evaluation and feedback to the senior colleagues.

Fifth, several participants expressed a willingness to educate and learn. To identify this behavior early and train young doctors in different feedback methods would allow for a more open environment in the future. To be able to maintain the knowledge and regular use the feedback methods taught would be essential, perhaps by early supervising interns or set up goals for regular feedback conversations with the supervisor after the feedback training.

### Methodological considerations

There are both strengths and limitations in this study. The coding was conducted by two researchers separately and additional researchers read the interviews and were involved in discussion of the codes and formation of the themes to heighten credibility. The transferability of the results is enhanced by the integration of multiple surgical specialties, inclusion of both university and regional hospitals, and data collection from over four years. The inclusion of participants not receiving employment was purposefully done to reflect a true recruitment process. As the responses did not reflect a specific clinic or department and applicants came from different hospitals, the results are likely generalizable to other specialties as well. However, not being the aim of this study investigation of potential differences between specialties could be an aim for future research. A limitation of the study is that it did not examine in detail how long the participants have worked as a doctor prior to their application. Participants with a more extensive background in the medical field may have yielded more nuanced responses and detailed scenarios which could impact the data and therefore the results. For example, being a specialist in another field likely means greater experience, and presumably more confidence that would have an impact one's response to any feedback.

Decisions regarding the size of the study population were guided primarily by the requirements of the quantitative methods within the overarching, longitudinal project. However, to evaluate the sufficiency of participants, we have applied the concept of information power as described by Malterud et al. [41] In this study, having a broad, exploratory aim, and a cross-case analysis using a specific cohort of applicants for a surgical position, our material could be considered adequate.

## Conclusion

This study revealed barriers to an effective feedback culture across surgical departments and disciplines through the perspectives of a pool of applicants for a residency or locum position.

A culture of reluctance to give feedback was a strong and common denominator. Dependent of the feedback one had received, and sometimes unfair critique, a common description was to filter and analyze before possibly accepting it. This processing of feedback could possibly be a method of survival and thriving in the contemporary workplace and be part of supervisory meetings. Unfortunately, many participants described themselves to be reluctant in providing feedback and criticism, a sign that the culture is deeply rooted. Suggestions and initiatives to change the feedback culture are proposed, which include change of scheduling, pairing with supervisors for longer periods, formative feedback crossing hierarchical levels both ways, and educational training for all levels.

## CRediT authorship contribution statement

**Hanne Pedersen:** Writing – original draft, Formal analysis, Conceptualization. **Alexander Tejera:** Writing – review & editing, Formal analysis, Conceptualization. **Christopher Mathieu:** Writing – review & editing, Methodology, Investigation, Conceptualization. **Britt-Marie Johansson:** Writing – review & editing, Methodology, Investigation, Conceptualization. **Magnus Anderberg:** Writing – review & editing, Methodology, Investigation. **Kristine Hagelsteen:** Writing – review & editing, Methodology, Investigation, Conceptualization.

## Ethics approval

Ethical approval for the longitudinal research project, including the study of this article, was obtained from the Swedish Ethical Review Agency (approval number 2016/1050, with amendments 2022-04361-02, and 2023-04850-02).

## Declaration of Generative AI and AI-assisted technologies in the writing process

During the preparation of this work HP used DeepL Write Pro in order to improve the language and readability. After using this tool/service, all authors reviewed and edited the full manuscript and take full responsibility for the content of the publication.

## Funding

This work was supported by *Prostatacancerförbundet* and *Löf* (regionernas ömsesidiga försäkringsbolag).

## Declaration of competing interest

The authors declare the following financial interests/personal relationships which may be considered as potential competing interests: Kristine Hagelsteen reports financial support was provided by Prostatacancerförbundet. Kristine Hagelsteen reports financial support was provided by Löf (regionernas ömsesidiga försäkringsbolag). If there are other authors, they declare that they have no known competing financial interests or personal relationships that could have appeared to influence the work reported in this paper.

## Appendix A Supplementary data

Supplementary material Image 1
