# Supplementary material for: Feedback gap and strategies for handling criticism in early surgical career
Source: Surg Open Sci. 2025 Nov 21;28:81–8. doi: 10.1016/j.sopen.2025.11.005 (PMC12702254; doi:10.1016/j.sopen.2025.11.005)
Supplement: Supplementary file 1 — Supplementary material [file mmc1.docx]

**Appendix 1**

**Interview Guide**
Letters in heading and brackets show the theme the questions are probing. A positive description that contrasts to the negative description of problem domains, is used to be in line with traditional known areas of competence. Motivation (M), Empathy (E), Communication (Cm), Self-assurance and Flexibility (SF), Attention (A), Leadership (L), Lifelong Learning (LL), Cooperation and Teamwork (Co) and Problem Solving (PS).

Introduction

1. Why do you want to become a surgeon? Your goal? (M)

2. What makes you feel good at work? (SF)

3. What makes you feel bad at work? (SF)

4. What are you good at? What are your weaknesses? (SF)

5. What is the biggest failure you have experienced in your job? (SF)

Understanding others (E)

6. Can you describe how you act when you rst meet a patient? What is the most important thing about that situation? (E)

7. What are the 3 most important sources you base your diagnosing upon? (PS)

8. How do you act when you feel you do not know how to proceed in treating a patient? (E)

9. If you have to give a negative message to a patient, how do you handle it? (Cm)

10. If your patient questions your assessment, how do you handle it? (SF)

Communication skills (Cm)

11. How do you ensure that important information you have about a patient reaches the right people?

12. How do you feel about supervising others? How do you give criticism? Give examples (C)

13. If you receive criticism from a colleague, how do you handle it? (SF)

Self-awareness/assurance? (SF)

14. Can you give an example of a situation where you quickly had to make a decisive decision in your job? How did you proceed?

(SF)

15. Have you been involved in an adverse event? Describe. What happened? How did you handle it? What did you learn from it?

(SF)

16. Can you give an example of a situation where you changed a contemplated action because of advice or recommendation

from someone else? What happened / what was the consequence? [When do you get help from others?] (SF)

Attention (A)

17. How do you handle situations where you have many tasks at once, for example in an emergency situation where you are

forced to leave the emergency room or ward for a few hours to go to surgery? (A) What do you do when you come back? (A)

Leadership (L)

18. Can you give examples of when and how you tried to exert in uence over a situation? (L)

19. Have you had a formal or informal leadership role during your professional or student time? How did you get these roles? How

are you as a leader? How is it expressed? (L)

Lifelong Learning (LL)

20. What do you think is the most important thing for maintaining skills in the role of surgeon? (LL)

Cooperation and teamwork (Co)

21. How would you describe the team that a surgeon is part of, the roles and responsibilities of these persons? (other specialists,

nurses, patients) Provide examples from reception, emergency or surgery (may not be possible through all 3?) (Co) Focus on 2 dif-

ferent roles to compare, for example, surgery and treatment teams before and after.

22. Have you experienced con icts in your workplace? Can you give examples? How did you act then? (Co)

23. What is collegiality for you? (Co)

24. If you witness that any of your colleagues act incorrectly, what do you do? For example, if you are assisting during surgery (Co)

Motivation (M)

25. How important is work in your life? (M) How do you cope with working nightshifts?

26. What do you think you will be doing in 10 years? (M)
